# Breastfeeding self-efficacy and postpartum depression: a cohort study

**DOI:** 10.1590/1518-8345.2110.3035

**Published:** 2018-09-06

**Authors:** Erika de Sá Vieira, Nathalia Torquato Caldeira, Daniella Soares Eugênio, Marina Moraes di Lucca, Isília Aparecida Silva

**Affiliations:** 1 PhD, Adjunct Professor, Escola Paulista de Enfermagem, Universidade Federal de São Paulo, São Paulo, SP, Brazil.; 2 Resident RN, Programa Multidisciplinar em Aleitamento Materno, Escola Paulista de Enfermagem, Universidade Federal de São Paulo, São Paulo, SP, Brazil.; 3 Prenatal Care Specialist, Escola Paulista de Enfermagem, Universidade Federal de São Paulo, São Paulo, SP, Brazil.; 4 Obstetrics and Gynecology Nursing Specialist, Instituto Israelita de Ensino e Pesquisa Albert Einstein, São Paulo, SP, Brazil.; 5 PhD, Full Professor, Escola de Enfermagem, Universidade de São Paulo, São Paulo, SP, Brazil.

**Keywords:** Postpartum Depression, Mental Health, Postpartum Period, Breast Feeding, Weaning, Self Efficacy

## Abstract

**Objective:**

to evaluate breastfeeding self-efficacy, the presence of postpartum depression symptons and the association between breastfeeding self-efficacy and postpartum depression with cessation of exclusive breastfeeding.

**Method:**

cohort study with 83 women. The instruments used were the Breastfeeding Self-Efficacy Scale and the Edinburgh Postnatal Depression Scale. Statistical analysis was conducted using the log-rank tests, analysis of variance and the Cox survival model.

**Results:**

breastfeeding self-efficacy (p = 0.315) and postpartum depression (p = 0.0879) did not show any statistical difference over time. The chances of cessation of exclusive breastfeeding decreased by 48% when self-efficacy changed from low to medium and by 80% when it changed from medium to high. Postpartum women who scored ≥10 on the Edinburgh Postnatal Depression Scale interrupt exclusive breastfeeding, on average, 10 days earlier than those with a score ≤9, whose median breastfeeding duration was 38 days postpartum.

**Conclusion:**

breastfeeding self-efficacy was proved to be a protective factor for exclusive breastfeeding, while postpartum depression is a risk factor.

## Introduction

The practice of exclusive breastfeeding for 6 months and mixed breastfeeding up to 2 years of age or beyond is considered the single most effective intervention in public health and could prevent, annually, the death of six million children under one year of age, since it protects against gastrointestinal and respiratory infections, sudden infant death syndrome, obesity and malnutrition^1^ ^-^ ^2^. However, the global average of exclusive breastfeeding (EBF) in children under six months of age is 36%, and improving this rate is a national and international challenge^2^.

The II Breastfeeding Prevalence Survey in Brazilian Capitals and in the Federal District showed that the mean of exclusive breastfeeding in children under six months of age in the Brazilian capitals is 41%, with higher rates in the North region (45.9% %), followed by the Center-West (45%), South (43.9%), Southeast (39.4) and Northeast (37%)^3^. 

Mother’s confidence in her ability to breastfeed her child has been shown as an important protective factor in the practice of exclusive breastfeeding, since women with a high level of breastfeeding self-efficacy demonstrate greater effort and persistence to overcome the possible difficulties, which are interpreted as challenges, not as reason for discouragement. Women with low self-efficacy are three times more likely to discontinue breastfeeding early^4^ ^-^ ^6^. 

Low level of education, first pregnancy, low number of prenatal consultations, no partner, intra and/or interpersonal conflicts, low or moderate pain and postpartum depression are considered risk factors for breastfeeding self-efficacy, while breastfeeding in the first hour of life, decision to breastfeed made during pregnancy, prior breastfeeding experience and social support are protective factors^4^ ^,^ ^7^ ^-^ ^10^.

Maternal mental health, especially depression, has a major impact on the health of women and children^11^. It is estimated that 5.2-32.9% of women experience depression during pregnancy and 4.9-59.4% have postpartum depression, depending on the criteria and parameters adopted for diagnosis and identification^12^. Evidence shows that, when they are not identified and properly treated, symptoms of depression can last for months or even years after delivery, increasing the risk of maternal suicide^12^ ^-^ ^13^.

Postpartum depression (PDD) can be characterized by irritability, anhedonia, anxiety, persistent discouragement, guilt, among other symptoms. These symptoms generally begin between the fourth and sixth week postpartum and have an impact on the physical and mental well-being of the woman, the health of the infant and the relationship between them^14^ ^-^ ^15^.

Risk factors for PPD are low socioeconomic status, intra- and interpersonal conflicts, and stressful life events, such as health problems and complications in pregnancy, childbirth, postpartum and breastfeeding^11^.

Studies show that women with PPD have less positive interaction with their children, a more negative perception about the child’s behavior, an increased risk of early interruption of EBF and a positive association with child malnutrition^5^ ^,^ ^11^ ^,^ ^16^ ^-^ ^17^.

The association between breastfeeding and maternal mental health has been confirmed in several studies, despite the inconclusive results regarding the cause-effect relationship. The difficulties in breastfeeding and in the weaning process are some of the causes for the increase in the rates of postpartum depression, confirming the positive influence of breastfeeding in the reduction of PPD symptoms. However, there is a high probability of breastfeeding being affected by postpartum depression^5^ ^,^ ^18^ ^-^ ^20^.

Considering the relevance of breastfeeding and postpartum mental health for improved rates of infant and maternal morbidity, the following research question was formulated: “Are breastfeeding self-efficacy and postpartum depression symptoms associated with the interruption of exclusive breastfeeding?”.

To answer this question, the present study aimed to evaluate breastfeeding self-efficacy, the presence of postpartum depression symptoms and the association of breastfeeding self-efficacy and postpartum depression with the interruption of exclusive breastfeeding.

## Method

This is a prospective cohort study conducted at the Incentive and Support Center for Breastfeeding and Human Milk Bank, linked to the Federal University of São Paulo, located in the city of São Paulo. 

The sample size was calculated considering a 20% incidence of PPD^21^, a 9% error and a 95% confidence limit, resulting in a minimum of 76 participants. The inclusion criteria were women who were exclusively breastfeeding their infants. The exclusion criteria were women who were more than 60 days postpartum at the time of the first interview.

In the period from July 2013 to October 2015, 208 women who were followed up at the breastfeeding clinic accepted to participate in the study by signing the Informed Consent Form. Follow-up was maintained until April 2016, with 83 women followed up for 210 days postpartum, the period defined as cutoff for the analysis of data in this study. The other 125 withdrew themselves from the long-term follow-up, but remained long enough to compose the previous cross-sectional study sample^5^.

In order to prevent early weaning, the breastfeeding clinic conducts monthly consultations, or at shorter intervals when there are complications with the mother-child dyad. The interviews for data collection occurred monthly in the clinic when these women went for their breastfeeding consultations and were conducted by two health professionals previously trained by the researcher. In case of unavailability to respond to the instruments during their stay in the service, an e-mail was sent with an access link to respond to the instrument via Internet, or telephone interviews were conducted on a day and time previously scheduled. The calls were recorded and the responses transcribed to the instruments by the interviewer. 

Socio-demographic data (age, level of education, occupation, civil status and family income), obstetric history (gravidity and parity, number of prenatal consultations in the current gestation and complication in the current gestation, delivery and/or postpartum), characteristics of the breastfeeding practice (history of breastfeeding, type of breastfeeding and complications in current breastfeeding) and characteristics related to intra and interpersonal relationships (history of violence, complaints about herself, her current partner and/or her child, and satisfaction with marital and family relationships established during their postpartum period) were collected from the medical records and recorded in an instrument developed specifically for this study.

The instruments used to identify breastfeeding self-efficacy and screen for PPD symptoms were the Breastfeeding Self-Efficacy Scale (BSES) and the Edinburgh Postnatal Depression Scale (EPDS), all validated for Brazil^6^ ^,^ ^22^.

The BSES and EPDS scales were applied in up to seven different moments, according to the number of days after delivery: 0 to 30 days, 31 to 60 days, 61 to 90 days, 91 to 120 days, 121 to 150 days, 151 to 180 days and 181 to 210 days.

The EPDS^22^ is a Likert-type scale, composed of 10 statements about the severity or duration of symptoms experienced or not in the week preceding the test application. The EPDS score range from 0 to 30 points, with 10 being the nationally recommended cut-off score for DPP symptom screening.

The BSES is also a Likert-type scale with a total of 33 items evaluating two categories: Technique and Intrapersonal Thoughts. The first category is composed of 20 items referring to the technical management of breastfeeding. The second one, composed of 13 items, evaluates the desire, motivation and satisfaction of the woman regarding this practice. For each item investigated, there is a score ranging from 1 (totally disagree) to 5 (totally agree). The total score ranges from a minimum of 33 to a maximum of 165, classifying breastfeeding self-efficacy at three levels: low (33 to 118 points), medium (119 to 137) or high (138 to 165)^23^ ^-^ ^24^.

The socio-demographic, clinical and relationship characterization data were analyzed descriptively. In order to assess breastfeeding self-efficacy and postpartum depression in the period studied, as well as to analyze the association of time until interruption of exclusive breastfeeding with breastfeeding self-efficacy and postpartum depression symptoms, the log-rank test was used to compare the survival curves (Kaplan-Meier) and the Cox survival model was used for the multivariate analysis.

The Cox proportional hazards survival model was adopted. The BSES and EPDS were considered as covariates and measured at different moments to increase the accuracy of PPD incidence estimates, and data from all women who were followed up for less than 210 days and did not have depression were included. The model considered the time of breastfeeding until the interruption of the EBF, as well as the occurrence of censoring when it was not possible to observe the interruption of EBF during the follow-up time.

To evaluate if the values ​​of the scales were different during the seven moments of application, the non-parametric ANOVA with repeated measures was carried out, considering only the time (moments of application) as a factor.

The software used for data analysis was R 3.1.2, and the level of significance adopted for all analyzes was 0.05.

This is an investigation extracted from the research “The interface between the experience of postpartum depression symptoms and the breastfeeding process”, linked to the Postdoctoral Program of the Nursing School of the University of São Paulo in 2016. The study was registered in the Brazil Platform under the number of the Certificate of Presentation for Ethical Appreciation (CAEE) 14507113.9.0000.5392.

## Results

The 83 women in postpartum were, on average, 30 years old. Regarding level of education, 41.55% completed secondary education and 40.58% had higher education. In addition, 50.72% had formal employment and 54.68% had a family income of 1 to 3 minimum wages. The majority (86.96%) of the women lived with their partner, the mean of pregnancies was 2.26, and 97.6% of the participants had more than six prenatal consultations. Complications in gestation, delivery and postpartum were present in 51.92%, 18.05% and 18.84% of the cases, respectively.

Among the women in postpartum, 38.12% had previous breastfeeding experience and, in the first 60 days after delivery, 62.14% had some kind of complication in breastfeeding.

Of these women, 19.61% reported they had been victims of some type of violence during their lives. History of psychiatric disorders was present in 30.39% of the sample, of which 27.32% were related to a previous episode of depression. The majority of the women in postpartum had no complaints related to themselves (66.83%), the infant (88.29%) or the partner (86.22%). The marital relationship improved after the birth of the baby for 53.06% of the women. The perception about the relationship with family members was satisfactory in 87.75% of the cases.

**Figure 1:**
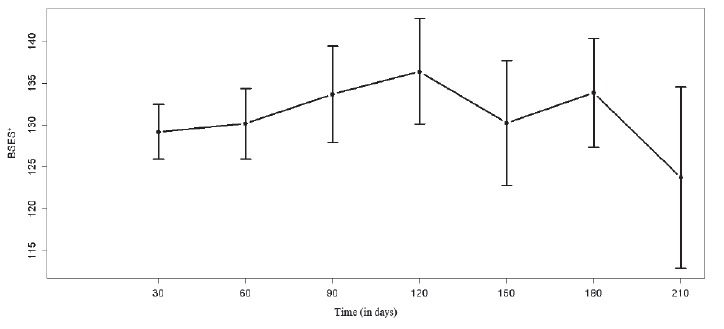
Breastfeeding self-efficacy during follow-up of 30-210 days postpartum (mean and 95% confidence interval). São Paulo, SP, Brazil, 2013 to 2016.

**Figure 2:**
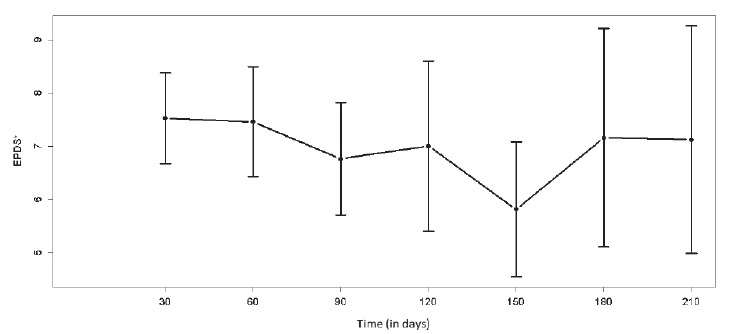
Scores of postpartum depression symptoms during follow-up of 30-210 days postpartum (mean and 95% confidence level). São Paulo, SP, Brazil, 2013 a 2016

The levels of breastfeeding self-efficacy (p = 0.315) and the presence of PPD symptoms (p=0.0639) over time, shown in the graphs in Figures 1 and 2, did not show statistical difference over the months.

*Breastfeeding Self-Efficacy Scale

*Edinburgh Postnatal Depression Scale

The analysis of the time until the interruption of EBF, ranging from 0 days to 210 days postpartum was divided according to the following aspects: general, BSES and EPDS levels, and Cox model to relate the time until the interruption of EBF with the EPDS and BSES scales.

The interruption of the EBF was accentuated during the first 60 days, followed by a slight decline after this initial period. The median was 36 days postpartum.

**Figure 3:**
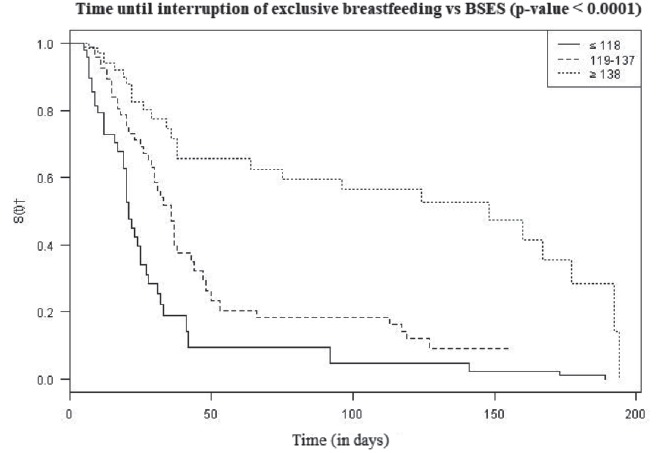
Time until interruption of exclusive breastfeeding according to breastfeeding self-efficacy levels. São Paulo, SP, Brazil, 2013 a 2016

The analysis of the time until interruption of EBF according to BSES levels (Figure 3) showed that there was an earlier interruption in the group of women with ≤118 points on the BSES scale. The median time of weaning for this group was 21 days postpartum, whereas the median time for the groups with scores from 119 to 137 and ≥138 was 36 and 148 days, respectively, presenting statistical difference (p-value <0.0001) when we apply the Cox model only with the BSES scale.

*Breastfeeding Self-Efficacy Scale; † Student’s t-test

**Figure 4:**
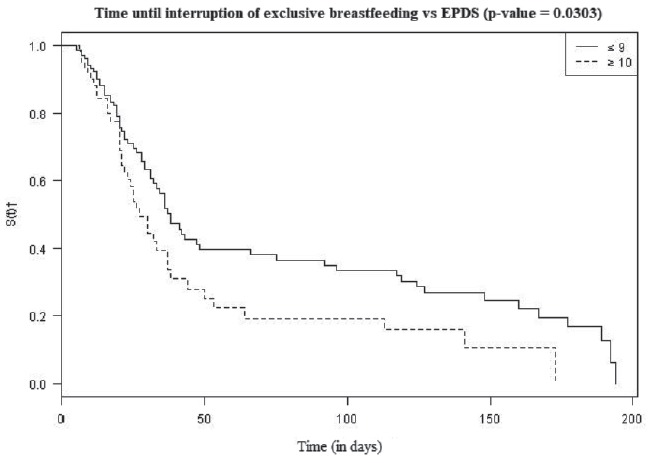
Time until interruption of exclusive breastfeeding according to scores of the Edinburgh Postnatal Depression Scale. São Paulo, SP, Brazil, 2013 a 2016

The analysis of the time until interruption of EBF according to the EPDS evidenced the existence of an earlier interruption for the group of women with ≥10 points in the EPDS scale. The median in the group with ≤ 9 points was 38 days and in the group with ≥ 10 points it was 27 days postpartum. The time until interruption in the two groups (Figure 4) demonstrated statistical difference (p-value <0.0303) when we applied the Cox model only with the EPDS scale.

*Edinburgh Postnatal Depression Scale; † Student’s t-test

The results from the Cox proportional hazards model regarding time until the interruption of EBF and the BSES and EPDS scales measured over time, show that, in the presence of the BSES scale, the EPDS is not statistically significant ( p = 0.9076). The chance of interruption of EBF when the range of ≤118 to 119 changes to 137 decreases by 48% (p = 0.0058) and, if it changes to ≥138, the chances decrease by 80% (p <0.01001). The assumption of proportional hazards was verified, but there was not enough evidence to refute it. 

## Discussion

Knowing fragilities’ aspects in mothers’ perceptions about their ability to breastfeed is of paramount importance for the establishment and maintenance of breastfeeding, since these aspects can be modified^25^ ^-^ ^26^. 

The increase in levels of breastfeeding self-efficacy in the first 120 days after delivery, identified in the present study and compatible with the literature, may be related to the adaptation of women in postpartum to the needs of their infants^26^. When they feel safer and less anxious in the maternal function, women become more confident in their ability to interpret and solve difficulties in breastfeeding, which were experienced by 62.14% of the women investigated. This result is compatible with the scientific literature, which indicates that 60% to 80% of women in postpartum experience complications in breastfeeding^27^ ^-^ ^29^.

The decrease in breastfeeding self-efficacy levels 120 days postpartum may be related to the return of these women to work. Considering that 50.72% of the women in this study had formal jobs and were on 120-day maternity leave, this data reinforces the negative impact of returning to work on the maintenance of EBF^4^ ^,^ ^25^. 

The association between self-efficacy levels and duration of breastfeeding demonstrated a faster interruption of EBF in postpartum women with low self-efficacy (median of 21 days). On the other hand, those with medium and high self-efficacy levels showed a median of 36 and 148 days of EBF, respectively. Similar results were found in other studies^18^ ^,^ ^25^ ^,^ ^30^, reinforcing the need for early identification and continuous evaluation of aspects that are more difficult for women throughout the breastfeeding process, aiming to implement individual and effective interventions to increase their confidence in the role of nurture.

In this study, the chance of interruption of EBF significantly reduced with increasing breastfeeding self-efficacy, decreasing by 48% when moving from low to medium self-efficacy and by 80% when moving from medium to high. Considering that global rates of initiation and maintenance of breastfeeding, more specifically exclusive breastfeeding, are unsatisfactory and that 53% of children under six months are not exclusively breastfed in low-income countries, 61% in lower-middle-income countries and 63% in upper-middle-income countries, the results of the present study are relevant^2^. 

The relationship between breastfeeding and symptoms of postpartum depression seems to be influenced by the woman’s intention, during pregnancy, to breastfeed after the child is born. A study that investigated women from gestation until up to 32 weeks postpartum found that non-depressed women who planned to breastfeed were less likely to develop postpartum depression when they were successful in breastfeeding. Thus, not being able to breastfeed as expected increases the risk of PPD^16^.

Other studies indicate that the presence of depressive symptoms in the postpartum period increases the chances of mother-child dyad difficulties in breastfeeding, decreasing maternal confidence in breastfeeding, and increasing the probability of interruption of EBF, confirming the findings of the present study^10^ ^,^ ^18^ ^-^ ^19^ ^,^ ^25^ ^,^ ^28^.

The interruption of EBF analysis and its association with PPD revealed that postpartum women with ≥ 10 points in the EPDS abandon exclusive breastfeeding on average 10 days before those with ≤ 9 points. Research shows that women with depressive symptoms during pregnancy are more likely to early introduce milk formula in the infant’s diet and that an one-point increase in the EPDS score in the immediate postpartum period increases by 6% the odds of weaning infants between 12 and 14 weeks of their life, a period identified as critical for cessation of EBF in the present study^25^ ^,^ ^31^.

The results of this research reinforce evidence about the importance of considering the mental health of women and its association with breastfeeding self-efficacy as a strategy to improve the global rates of maternal and child morbidity. In this sense, the continuous application of the BSES and the EPDS was implemented in the breastfeeding clinic studied, with adherence of the multi-professional team, allowing the monitoring of the confidence levels of the postpartum women for maintaining exclusive breastfeeding, and providing early identification and possibility of referral for the definition of appropriate therapy for women with PPD.

As limitations of this study, the difficulties of prolonged follow-up can be highlighted, since many women withdrew themselves from the long-term follow-up, usually after 120 days, which raises the possibility of using telephone follow-up as a strategy to identify eventual demands and support interventions for this group. In addition, the purposes of this research did not include women in the gestation period, variables regarding other types of breastfeeding besides EBF and clinical diagnosis of PPD. Future research is necessary to deepen the knowledge about the possible associations between the breastfeeding process and the variables mentioned above with depression, from pregnancy to twelve months postpartum. 

## Conclusion

The results show that the chances of cessation of EBF decrease by 80% when breastfeeding self-efficacy changes from medium to high, and by 48% when it changes from low to medium, which demonstrates the existence of a positive association with exclusive breastfeeding. The presence of PPD symptoms presented a statistically significant association (p-value <0.0303) with the cessation of EBF. However, no association was found between the self-efficacy measured by the BSES and the symptoms of PPD measured by the EPDS in the group studied. Breastfeeding self-efficacy and PPD remained the same in the periods evaluated. Therefore, breastfeeding self-efficacy has been proved to be a protective factor for exclusive breastfeeding, while postpartum depression is a risk factor.
